# Facultative scavenging feeding habits in *Acanthurus chirurgus* (Bloch, 1787) (Acanthuriformes: Acanthuridae)

**DOI:** 10.3897/BDJ.8.e53712

**Published:** 2020-07-27

**Authors:** Benjamín Delgado-Pech, Antonio Almazán-Becerril, Jorge Peniche-Pérez, José Adán Caballero-Vázquez

**Affiliations:** 1 Centro de Investigación Científica de Yucatán A.C. (CICY), Unidad de Ciencias del Agua, Q. Roo, Mexico Centro de Investigación Científica de Yucatán A.C. (CICY), Unidad de Ciencias del Agua Q. Roo Mexico

**Keywords:** Caribbean Sea, coral reef, herbivorous fish, Mesoamerican Reef System.

## Abstract

The family Acanthuridae is a key component of coral reef ecosystems as it controls macroalgae biomass buildup. During routine monitoring of benthic communities in the Mexican Caribbean, we observed unusual behaviour of a group of *Acanthurus chirurgus*, which were feeding on a tuna head left on site by travel tour personnel. This phenomenon has been documented in other herbivorous fish species, especially in places where tourism is a major coastal activity. Although many *Acanthurus* seek additional sources of protein by feeding on detritus, it is unusual for them to feed directly on fish flesh. *Acanthurus chirurgus* will incorporate proteins from animal tissues whenever the opportunity arises. Such opportunities occurred rarely in the past, but have become more frequent recently, related to increasing tourism activities where flesh is used as bait to attract the surrounding fauna.

## Main text

**Figure 1.**
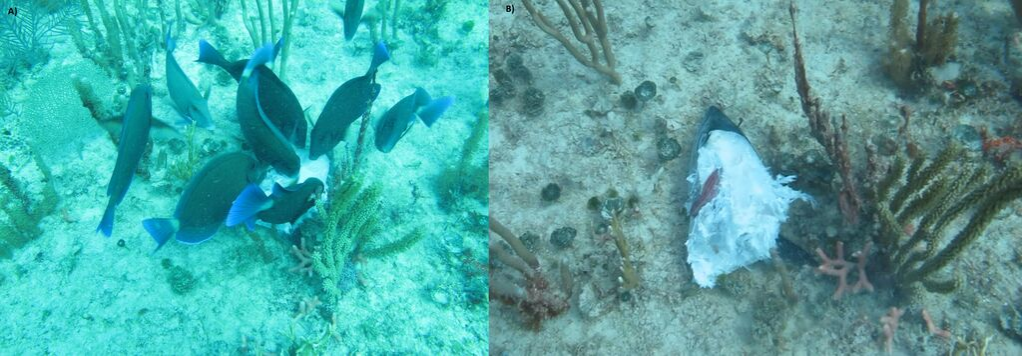
Two frames of the video showing surgeonfish tearing apart a tuna head (A). The fish head showing evidence of the surgeonfish bites (B).

Acanthuridae is a conspicuous and abundant family of herbivorous fish commonly inhabiting coral reef systems. Currently, 86 species in six genera are recognised to belong to this family, whose distribution encompasses tropical and subtropical coastal environments (ITIS 2020). Three species in the genus *Acanthurus* Forsskål, 1775 are easily identified along the Caribbean basin: *Acanthurus coeruleus* Bloch & Schneider, 1801, *A. bahianus* Castelnau, 1855 and *A. chirurgus* (Bloch 1787) (Duarte and Acero 1988). These species play a fundamental role in the functioning of coral reef ecosystems due to their capability to control macroalgae abundance (Ogden and Lobel 1978). During routine monitoring of benthic communities in coral reef patches in Playa del Carmen, Mexico (Mesoamerican Reef System) in November 2019, we observed a group of surgeonfish (*Acanthurus chirurgus*) feeding on a tuna head, possibly *Euthynnus* sp. (Fig. 1, *Matt. Suppl Fig. 2*). The approximately 4.5 m-deep site is frequently visited by numerous snorkeling groups. Fish flesh is commonly used in fishing and diving activities as bait to attract fish and sharks.

This behaviour demonstrates that this species exhibits facultative feeding habits and raises several questions: 1) How widely does this facultative behaviour occur amongst herbivorous reef fish families? 2) what physiological or ecological mechanisms trigger facultative scavenging habits in *Acanthurus*? and 3) what are the implications of this behaviour for the trophic structure and functioning of these ecosystems?

Facultative feeding habits have been reported in numerous groups of herbivorous fish. For example, Drew and McKeon (2019) observed many non-carnivorous fish species, including *Acanthurus*, eating small fragments of fish flesh (tuna heads) used to attract sharks during diving tourism activities in Fiji. Duarte and Acero (1988) found crustaceans, remains of soft corals, as well as fish scales and eggs, in the stomach contents of *A. chirurgus* specimens from the Colombian Caribbean; they also found calanoid copepods in *A. coeruleus* specimens. This implies that this genus also exhibits facultative zooplanktophagous habits, especially during seasonal scarcity of macroalgae. Choat et al. (2002), Choat et al. (2004) showed that only a few of the 17 species from the Great Barrier Reef analysed were strict herbivores and that most included planktonic animal matter, sediment and detritus in their diet, in addition to macroscopic algae. Ontogenetic changes and reproductive stages may trigger the shift in feeding behaviour in numerous nominally herbivorous fish. Herbivores must maintain a high ingestion rate because of the low nutrient assimilation efficiency (Ogden and Lobel 1978). Then, during periods of rapid growth and reproduction, the demand for nutrients (mainly proteins) increases and can only be met by feeding on animal matter. For example, Johnson et al. 2017 found that juveniles of the herbivorous fish *Odax pullus* J. R. Foster 1801 consumed more animal matter (crustaceans) than adults to meet the protein demand required by their fast growth rate. Studies evaluating the dietary habits of the three species of *Acanthurus* occurring in the West Atlantic agree that *A. chirurgus* and *A. bahianus* share a similar diet, different from that of *A. coeruleus* (Clavijo 1974, Duarte and Acero 1988, Tilghman et al. 2001, Thelma et al. 2001). The latter depends to a greater extent on non-coralline red algae (such as *Acanthophora spicifera*), whereas *A. chirurgus* and *A. bahianus* are less selective and include a broader range of available resources in their diet (Dromard et al. 2015). Some *Acanthurus* species likely possess the physiological mechanisms to digest and assimilate flesh, which could be interpreted as a response to the continued search of additional protein sources given its low availability in coral reefs (Dromard et al. 2015, Mendes et al. 2018). In this context, *A. chirurgus* will incorporate protein from animal tissues as the opportunity arises. Such opportunities were probably less common in the past, but have now become more frequent as coastal tourism activities which use fish flesh to attract the surrounding fauna have increased in recent years as a result of the sustainable tourism approach and as tour operators conduct activities aimed at preserving marine species.

## Figures and Tables

**Figure 2.** Video showing the shoals of surgeonfish tearing apart a tuna head.

## Hosting institution

Centro de Investigación Científica de Yucatán A.C. (CICY). Unidad de Ciencias del Agua. Calle 8, No. 39, Mz. 29, S.M. 64, 77500. Cancún Quitana Roo. México.
